# Laparoscopic Ladd’s procedure for intestinal malrotation in small infants with midterm follow-up

**DOI:** 10.1186/s12876-023-03046-1

**Published:** 2023-11-20

**Authors:** Xuepeng Zhang, Lvna Xiang, Tong Qiu, Jiangyuan Zhou, Guowei Che, Yi Ji, Zhicheng Xu

**Affiliations:** 1 Department of Pediatric Surgery, West China Hospital, Sichuan University, Chengdu, 610041 China; 2 Department of Critical Care Medicine, West China Hospital, Sichuan University, Chengdu, 610041 China; 3 West China School of Nursing/West China Hospital, Sichuan University, Chengdu, 610041 Sichuan China; 4 Department of Thoracic Surgery, West China Hospital, Sichuan University, Chengdu, 610041 China

**Keywords:** Malrotation, Infants, Laparoscopic, Ladd’s procedure

## Abstract

**Background:**

The objective of this study was to evaluate the safety and efficacy of laparoscopic Ladd’s procedure (LL) for intestinal malrotation (IM) in small infants.

**Methods:**

All patients aged < 6 months with IM who underwent Ladd’s procedures between January 2012 and December 2019 were enrolled. The perioperative demographics and midterm follow-up results were retrospectively reviewed and compared between patients who underwent LL and open Ladd’s operation (OL).

**Results:**

Fifty-five patients were enrolled for analysis. The baseline characteristics were well matched in the two groups. The rate of volvulus was similar in the two groups (76.2% vs. 73.5%, *P* = 0.81). Two cases in the LL group were converted to OL due to intraoperative bleeding and intestinal swelling. The operative time (ORT) was not significantly different between the two groups (73.8 ± 18.7 vs. 66.8 ± 11.6 min, *P* = 0.76). Compared to the OL group, the LL group had a shorter time full feed (TFF) (3.1 ± 1.2 vs. 7.3 ± 1.9 days, *P* = 0.03) and a shorter postoperative hospital stay (PHS) than the OL group (5.5 ± 1.6 vs. 11.3 ± 2.7 days, *P* = 0.02). The rate of postoperative complications was similar in the two groups (9.5% vs. 11.8%, *P* = 0.47). The LL group had a lower rate of adhesive obstruction than the OL group, but the difference was not significant (0.0% vs. 11.8%, *P* = 0.09). One patient suffered recurrence in the LL group, while 0 patients suffered recurrence in the OL group (4.8% vs. 0.0%, *P* = 0.07). The rate of reoperation in the two groups was similar (4.8% vs. 8.8%).

**Conclusions:**

The LL procedure for IM in small infants was a safe and reliable method that had a satisfactory cosmetic appearance and shorter TFF and PHS than OL.

## Introduction

Intestinal malrotation (IM) is a rare congenital intestinal anomaly with an incidence of 0.2-1% in the pediatric population [1]. It results from errors in fetal intestinal rotation and fixation. Without proper treatment, IM can result in fatal consequences such as midgut volvulus. The open Ladd’s procedure (OL), described by William E. Ladd in 1936, is the optimal treatment in symptomatic patients with IM [2, 3]. However, long-term follow-up studies revealed that postoperative complications frequently occurred in children with IM who received OL [4, 5]. Intestinal obstruction was observed in one in four of the patients after OL treatment [4, 5].

Since being developed in 1995, the laparoscopic Ladd’s procedure (LL) has been increasingly used for the treatment of IM with the advancement of minimally invasive surgical (MIS) techniques [6–17]. Although several studies reported that the incidence of postoperative complications was lower in patients treated with LL, the benefit of LL is still controversial [18, 19]. In comparison with OL, LL required a longer operation time and had a higher incidence of postoperative volvulus in previous studies [18, 20]. Furthermore, evidence regarding the safety and efficacy of LL in small infants is lacking since the laparoscopic approach is more commonly performed in older children with IM [17, 21–25]. We conducted a retrospective study to evaluate the safety and efficacy of LL in small infants aged < 6 months in our hospital.

## Materials and methods

### Design and study population

This was a retrospective study of patients with IM aged < 6 months who underwent open or laparoscopic Ladd’s procedure between January 2012 and December 2019. This study was conducted according to the Declaration of Helsinki and was approved by the Ethics Committee of West China Hospital. All parents of the enrolled children were informed and provided written informed consent. Patients’ parents were given the option to choose the treatment (either OL or LL). Patients with symptoms of intestinal obstruction and intestinal ischemia who needed urgent Ladd’s procedure were excluded. Children who were lost to follow-up were also excluded from this study. The patients who underwent LL were assigned to the LL group, while patients who underwent OL were assigned to the OL group. In the present study, all the LL and OL procedures were performed by the same surgical team with a supervisor (ZCX). The data on perioperative demographics and midterm outcomes of follow-up were analyzed and compared between the LL group and OL group.

### Surgical technique

#### The procedure of OL

During the OL procedure, the patient was placed in a supine position. A transverse incision with a length of 5–8 cm was made above the level of the umbilicus. The remaining management was the same as traditional Ladd’s operations [3].

#### The procedure of LL

During the LL procedure, the patient was placed in a supine position with a monitor at the site of the patient’s head. The operator stood on the left side to the patient’s feet, and the camera assistant stood on the other. A 3 mm incision was made on the center of the umbilical ring with the open Hasson technique to establish the pneumoperitoneum at a pressure of 6–8 mmHg with a flow rate of 3–6 L/min. A 3 mm trocar and a 30° laparoscope were introduced into the peritoneal cavity. Under laparoscopic guidance, two 3 mm trocars were individually inserted at the bilateral midclavicular lines above the level of the umbilicus.

The procedure was well described in A. Suyodhan Reddy’s study [26]. Initially, diagnostic laparoscopy was performed using a 30°, 3 mm telescope. Principles of correction of malrotation as in open surgery were followed in all patients. Ladd bands were divided; the cecum and colon were released medially from the duodenum and pancreas. Duodenum and jejunal loops were straightened. The base of the mesentery was widened, and appendectomy was performed. The cecum and the colon were placed on the left of the midline and the straightened duodenum, and the small bowel was placed on the right side. Bowel fixation was not performed in Ladd’s Procedure.

### Data collection and statistical analysis

Data on perioperative clinical features were collected by reviewing the medical charts. The follow-up data were collected during outpatient clinic visits and/or using a telephone questionnaire. Prematurity was defined as gestational age < 37 weeks.

Data are expressed as the mean with SD. Student’s t tests and *chi-squared* tests were used to compare continuous and categorical descriptive variables, respectively. The software applied for statistical calculation was IBM SPSS 22.0 for Windows 10.0 (IBM Corp.). P < 0.05 was considered statistically significant.

## Results

### Data on perioperative demographics

There were 66 infants < 6 months with IM who underwent Ladd’s procedure between January 2012 and December 2019. Eleven patients were excluded from the study for the following reasons: urgent Ladd’s surgery in 6 (OL) and failure to follow-up in 5 (LL 3, OL 2). Finally, 55 patients were enrolled, of whom 21 had LL and 34 received OL. The distribution of age was similar in the two groups (35.6 ± 28.7 vs. 36.7 ± 23.3 months, *P* = 0.43). There were 11 males in the LL group and 14 males in the OL group (*P* = 0.37). No differences were found in the rate of prematurity and associated anomalies (Table 1).

**Table 1:** Preoperative characteristics of patients between two groups

	LL n = 21	OL n = 34	*P* value
Age, days	35.6 ± 28.7	36.7 ± 23.3	0.43
Male, n (%)	11 (52.4%)	14 (41.2%)	0.37
Prematurity, n (%)	5 (23.8%)	7 (20.6%)	0.17
Weight, kg	3.2 ± 0.8	3.4 ± 1.0	0.11
Associated anomalies, n (%)	8 (38.1%)	13 (38.2%)	0.19
Duodenum atresia	1 (4.8%)	2 (5.9%)	
Small intestinal atresia	4 19.0(%)	6 (17.6%)	
Cardiac anomalies	2 (9.5%)	3 (8.8%)	
Biliary tract anomalies	1 (4.8%)	1 (2.9%)	
Trisomy 21	0 (0.0%)	1 (2.9%)	

LL: laparoscopic Ladd’s procedure; OL: open Ladd’s operation

In the LL group, 16 cases of small bowel volvulus were found, while 25 cases of small bowel volvulus were found in the OL group (76.2% vs. 73.5%, *P* = 0.81). Two cases were converted to OL in the LL group due to intraoperative bleeding and intestinal swelling. The two cases were neonates aged 7 days and 9 days. One case was found to have intestinal swelling, which did not allow adequate space to perform LL. Another case bled near the Treitz ligament during the operation, which made unclear visualization of the operative field. The operative time (ORT) was not significantly different between the two groups (73.8 ± 18.7 vs. 66.8 ± 11.6 min, *P* = 0.76).

The LL group had a shorter time to full feed (TFF) than the OL group (3.1 ± 1.2 vs. 7.3 ± 1.9 days, *P* = 0.03). In addition, the LL group had a shorter postoperative hospital stay (PHS) than the OL group (5.5 ± 1.6 vs. 11.3 ± 2.7 days, *P* = 0.02). The rate of postoperative complications was not significantly different between the two groups (9.5% vs. 11.8%, *P* = 0.47) (Table 2).

**Table 2:** Intra- and postoperative demographics of patients between two groups

	LL n = 21	OL n = 34	*P* value
Small bowel volvulus, n (%) Conversion to open, n	16 (76.2%) 2 (9.5%)	25 (73.5%) *NA*	0.81 *NA*
ORT, minutes	73.8 ± 18.7	66.8 ± 11.6	0.76
Postoperative complication, n (%)	2 (9.5%)	4 (11.8%)	0.47
TFF (days)	3.1 ± 1.2	7.3 ± 1.9	0.03
PHS (days)	5.5 ± 1.6	11.3 ± 2.7	0.02

LL: laparoscopic Ladd’s procedure; OL: open Ladd’s operation; ORT: operative time; TFF: time full feed; PHS: postoperative hospital stay

### Midterm follow-up results

The follow-up time was 52.2 ± 31.8 months in the LL group and 53.8 ± 21.4 months in the OL group (*P* = 0.41). There were no cases of adhesive obstruction in the LL group, while there were 4 cases in the OL group (0.0% vs. 11.8%, *P* = 0.09). One patient suffered recurrence in the LL group, while 0 patients suffered recurrence in the OL group (4.8% vs. 0.0%, *P* = 0.07). There was no significant difference in the rate of reoperation between the two groups (4.8% vs. 8.8%, *P* = 0.55) (Table 3).

**Table 3:** The mid-term follow-up data of patients between two groups

	LL n = 21	OL n = 34	*P* value
Time of follow-up, months	52.2 ± 31.8	53.8 ± 21.4	0.41
Adhesive obstruction, n (%)	0 (0.0%)	4 (11.8%)	0.09
Recurrence, n (%)	1 (4.8%)	0 (0.0%)	0.07
Redo surgery, n (%)	1 (4.8%)	3 (8.8%)	0.55

LL: laparoscopic Ladd’s procedure; OL: open Ladd’s operation

## Discussion

Since LL was first developed in 1995 [27], this technique has been gradually adopted throughout the world due to the benefits of MIS. In one article comparing 53 cases (mean age of 4.4 years) of LL versus 86 cases (mean age of 0.3 years) of OL, Huntington showed that LL led to a significantly shorter length of stay than OL in the initial 30-day postoperative period [17]. This corresponds to the findings of Fraser [28] and Stanfill [6]; the mean length of stay in the LL group was 9 days compared with 16 days in the OL group. In our study, the median PHS was 5.5 days after LL, which was significantly shorter than 11.3 days (*P* = 0.02) after OL and similar to a prompt recovery from other reports [6, 17, 28, 29].

Although the literature is replete with articles and case series attesting to the safety and excellent outcome of the procedure [30–33], LL can be challenging when performed in small infants [11]. Some authors suggested caution when executing LL in neonates and infants younger than 3 months [19, 27], which probably stemmed from the tendency for increased conversion rates in smaller children. Hsiao reported a 50% conversion rate in neonates but only 18% in older patients [27], while Catania described a conversion rate up to 25.3% in their meta-analysis [18]. In another study [34], the overall conversion rate was 16% but increased to 19% when considering patients below 6 months of age and reached 37% in patients with a midgut volvulus. However, our case series showed that LL was feasible in small infants with a mean age of 35.6 ± 28.7 days. The rate of conversion to OL was 9.5%, which was advantageous over the other published series [11, 13, 28, 35]. An average operating time (ORT) of 73.8 min was seen in LL, which was comparable to 66.8 min (*P* = 0.76) in OL and comparable to other studies describing ORT for LL ranging from 53 to 120 min [6, 10, 11, 25, 28, 35]. The 9.5% of complications after LL was not significantly different from the 11.8% (*P* = 0.47) after OL, which was in a similar range as in other studies [10, 11, 22, 24, 25, 30, 35].

There were some studies in which both laparoscopy and laparotomy were compared regarding recurrent volvulus in elderly children. The first, by Fraser [28], found a postoperative recurrence of intestinal volvulus in six patients (2.4%). Remarkably, all six had an OL. In another study, Stanfill [6] found a higher percentage of volvulus of 6% after LL vs. 1% after OL. A third study by Hsiao and Langer [27] found no differences in the primary LL and primary or secondary OL, and neither had any recurrences. In other smaller series, where only laparoscopic data were collected, redo surgery rates ranged from 0 to 20% [11, 24, 34, 36]. However, in our study, during midterm follow-up, 1 case (4.7%) of recurrence and 0 cases of adhesive ileus occurred after LL, with 1 case reoperated, while 0 cases (*P* = 0.07) of relapse and 4 cases (*P* = 0.09) of adhesive ileus occurred after OL, with 3 (*P* = 0.55) reoperated.

Based on the above results, it is speculated that institutional factors, such as training opportunities and access to support, may play a role in the use of LL for small babies with IM. In addition, the experiences in laparoscopic surgery for small infants, such as thoracoscopic esophageal atresia repair and laparoscopic Kasai procedure, also helped surgeons quickly pass the learning curve of LL [37]. Nonetheless, the technique of LL applied in small infants with limited abdominal working space is still demanding, even for veterans. The two cases converted to OL in the LL group in this study were neonates aged 7 days and 9 days, suggesting a relatively small working space to perform the LL operation. Intestinal swelling in one case further narrowed the working space to perform LL, allowing inadequate working space. Local bleeding near the Treitz ligament in the other case made the visualization of the operative field unclear. Poor vision and local bleeding always contribute to inadequate straightening of the duodenum, which is the most frequent finding at reoperation [26]. The key to successful LM is accurate identification of the malrotated anatomy of the intestine and its mesentery and skillfully performing all steps of Ladd’s procedure for malrotation correction. Furthermore, the surgeon must explore the patency of the entire duodenum at the time of surgery because 28% of infants with duodenal atresia had malrotation and 19% of infants with jejunoileal atresia had malrotation, as reported by Vecchia [38].

It is important to note several limitations to the current study. First, this was a retrospective, nonrandomized, controlled study. Second, LL and OL procedures may be carried out by different surgeons from the same surgical team, and different surgical experiences may generate different results. Third, the failed follow-up data of a small number of patients might bias the final statistical calculations.

Despite the limitations mentioned above, LL in small infants with IM was a safe and reliable method in well-trained hands, which had satisfactory cosmetic appearances and shorter TFF and PHS than OL. The midterm results after LL were comparable to those after OL. LL can be regarded as an alternative option for small infants with IM.

## Conclusion

In this study, we compared the intra- and postoperative characteristics and midterm outcomes between small infants (younger than 6 months) with IM receiving LL and those receiving OL. LL did not lead to a significantly longer ORT. The LL group had shorter TFF and PHS than the OL group. The postoperative complications and midterm outcomes in the LL group were comparable to those after OL. LL was a safe and reliable method and could be regarded as an alternative option for small infants with IM.

## Data Availability

The data are available through the corresponding author.

## Abbreviations

LL: laparoscopic Ladd’s procedure
IM: intestinal malrotation
OL: open Ladd’s operation
ORT: operative time
TFF: time full feed
PHS: postoperative hospital stay
MIS: minimally invasive surgical
